# Treatment of cervical lymph node tuberculosis: When surgery should be performed? A retrospective cohort study

**DOI:** 10.1016/j.amsu.2020.05.006

**Published:** 2020-05-18

**Authors:** Adil Lekhbal, Kaoutar Chaker, Sara Halily, Reda Lah Abada, Sami Rouadi, Mohamed Roubal, Mohamed Mahtar

**Affiliations:** ENT Department, Face and Neck Surgery, Hospital August, 20’1953, University Hospital Centre IBN ROCHD, Casablanca, Morocco

**Keywords:** Lymph node tuberculosis, Surgical treatment, Paradoxical upgrading reaction, Drug resistance, Recurrence

## Abstract

**Background:**

lymph node tuberculosis is the most common form of extra pulmonary tuberculosis. Although diagnosis is usually difficult, therapeutic management remains a challenge and a subject of national and international debate.

**Materials and methods:**

the medical records of patients with cervical lymph node tuberculosis who were treated at 20 August Hospital, Casablanca, Morocco, between May 2017 and November 2018 were reviewed. The results of the treatment were analyzed retrospectively.

The aim of our work was to identify all causes of medical treatment failure in cervical lymph node tuberculosis, and to propose indications for the use of surgery in cervical lymph node tuberculosis in Morocco.

**Results:**

Out of a total of 104 patients, the mean age was 24 years, the sex ratio was 0.51 (women: 66.3%), twenty (19.2%) patients received medical treatment alone, and 84 (80.8%) patients required medical and surgical treatment. Surgery was required when the size of the lymphadenopathies was greater than or equal to 3 cm (p = 0.005), when the patient presented with an abscess and/or fistula(p = 0.005), when the patient presented with resistance to antibacillary drugs(p = 0.032), or developed a paradoxical upgrading reactions (p = 0.001), or when the patient had a recurrence of lymph node tuberculosis(p = 0.008) on multivariate analysis.

**Conclusion:**

antibiotic therapy remains the main treatment for all patients in lymph node tuberculosis, but the results of our work show the value of surgery in some indications.

## Highlights

• Our goal in this study was to identify causes of the failure of medical treatment in lymph node tuberculosis, to propose indications for surgery.
• The study has compared patients who were reported to be cured under medical treatment alone after a lymph node biopsy, and patients who required surgery after failure of medical treatment in lymph node tuberculosis.
• We recommend surgery when we have: adenopathies ≥3 cm in diameter, abscesses, and fistulas, recurrence, resistance to antibacillary drugs, and paradoxical upgrading reaction.

## Abbreviations

TB: tuberculosis
LNT: lymph nodes tuberculosis
WHO: World Health Organization
EPTB: extra pulmonary tuberculosis
PAS: paraaminosalicylate
IDSA: Infectious Diseases Society of America
ENT: Ear, Nose and Throat
MTB/RIF: *Mycobacterium tuberculosis*/rifampicin
LA: Lymphadenopathy
CI: confidence interval
N: number
OR: odds-ratio

## 1 Introduction

Tuberculosis (TB) is a major public health problem in the international community, the World Health Organization (WHO) estimated at 10.4 million cases in 2016 [1]. Extra pulmonary tuberculosis (EPTB) does not exceed 15% of all TB patients reported for many years [1,2]. However, there are some differences between WHO regions.

In Morocco, WHO estimates for the year 2016 that approximately 36,000 people have been affected by tuberculosis, with an estimated incidence of 103 cases per 100,000 persons [1]. Between 1980 and 2016, the proportion of reported EPTB cases increased from 23% to 46%, while the proportion of pulmonary TB cases decreased from 63% to 54% [3]. lymph nodes tuberculosis (LNT) is the most common form of EPTB, accounting for 37% of all reported EPTB cases in Morocco in 2016 [3].

Currently, LNT is treated with multidrug antibacillary chemotherapy [4]. However, in the 1970s, surgery was often necessary in the treatment of LNT, as the chemotherapy used, based on streptomycin, isoniazid, and paraaminosalicylate (PAS) for 3 years, did not give satisfactory results [5]. For this reason, ENT surgeons preferred surgery without medical treatment until the early 1990s [6], after which the WHO recommended treatment with rifampicin and isoniazid for 6 months and pyrazinamide for 2 months (WHO, 1997), this treatment was adjusted over the years by the WHO, and the number of publications regarding surgery for LNT has decreased considerably.

The Infectious Diseases Society of America (IDSA) recommends surgery for LNT only in unusual circumstances, but these are not explicit [7]. Although surgical excision combined with antibiotic therapy produces favorable results. We are not aware of any controlled studies that have compared the results of medical and surgical treatment with medical treatment alone [8].

In our context we find ourselves more and more in situations where patients retain some lymphadenopathies, or develop others despite well-conducted medical treatment.

The main goal of our study was to identify causes of the failure of medical treatment in LNT, and to propose indications for the use of surgery in cervical LNT in Morocco, and the secondary goal was to identify the epidemiological and clinical characteristics of our patients.

## 2 Materials and methods

We conducted a retrospective and analytical study in the ENT and cervico-facial surgery department at the 20 August hospital in Casablanca, for a period of 18 months (May 2017 to November 2018). The study was conducted and reported in line with STROCSS criteria [9].

Patients with cervical LNT confirmed on lymph node biopsy, by the presence of granulomatous inflammation with caseous necrosis, and/or Xpert MTB/RIF positivity with study of rifampicin resistance, and/or positive culture with study of antibacillary resistance, were included in the study. Patients received antibacillary multidrug therapy based on WHO recommendations in the treatment of LNT. While patients undergoing first-line surgical treatment outside of abscess and fistula, and those with poor adherence to medical treatment were excluded from the study.

The study has compared patients who were reported to be cured under medical treatment alone after a lymph node biopsy, and patients who required surgery after failure of medical treatment.

Patients were divided into 3 groups according to Lymphadenopathy (LA) characteristics (number of LA, as well as their size, presence of an abscess and/or fistula). Regarding the size of the LA, a diameter of 3 cm is used as a reference to differentiate between patients.

In this study, " cure " was defined as the complete disappearance of LA, abscesses, and fistulas after at least 6 months after the end of treatment. We analyzed the factors that required surgery in addition to medical treatment, comparing clinical and paraclinical data of patients who received medical treatment alone, and patients who required surgery after failure of medical treatment.

The main goal of our work was to identify causes of the failure of medical treatment in LNT, and to propose indications for the use of surgery in cervical LNT in Morocco, and the secondary goal was to identify the epidemiological and clinical characteristics of our patients.

The statistical analysis was performed with SPSS 23 software (SPSS Inc., Chicago, IL, USA), the statistical tests used were Chi-2 (or Fisher test when the theoretical counts were less than or equal to 5%) for the analysis of nominal variables, the analysis of ordinal variables, and quantitative variables was performed by Spearman correlations, A p < 0.05 was retained as the significant threshold with a 95% confidence interval (CI). A multivariate analysis by logistic regression was then conducted, taking as explained variabIe: “the use of surgical treatment after failure of medical treatment” and as explanatory variables ceIIes whose degree of significance was less than p < 0.20 in univariate anaIysis, from this initial muItivariate model, the variabIes whose adjusted degree of significance remained less than p < 0.05 were kept in the final model.

## 3 Results

During the study period, 104 cases of LNT were included in the study, the sex ratio was 0.51(women:66%), the mean age was 24 years, the age range between 20 and 40 years accounted for 51% of the cases, 27 patients had a recurrence of LNT, while 7 patients had multifocal tuberculosis, the duration of symptom progression was less than 2 months in 32.7% of the cases, and more than 2 months in 67.3% of the cases. Clinically, a single LA was found in 51.9% of cases, and multiple LA in 48.1% of cases, an abscess and/or fistula in 19 patients, the size of the LA was less than 3 cm in 34.6% of cases, and greater than or equal to 3 cm in 65.4% of cases, resistance to antibacillaries was found in 12 (11.5%) patients, the duration of medical treatment was 6 months in 76.9% of cases, and greater than or equal to 9 months in 23.1% of cases, during medical treatment, 24 (23.1%) patients developed a paradoxical upgrading reaction to antibacillaries (Table 1).

**Table 1:** Clinical and paraclinical characteristics of our patients.

Total number of patients	104 patients	
Age	Medium: 24 ans	
	maximum: 72 ans	
	Minimum: 3 ans	
	[20–40 years]: 51% of patients	
Gender	Male: n = 35 (33,7%)	
	Female: n = 69 (66,3%)	
History of LNT	n = 27 (26%)	
Multifocal tuberculosis	n = 7 (6,7%)	
Duration of symptom evolution	<2 month: n = 34 (32,7%)	
	≥2 month: n = 70 (67,3%)	
Number of LA	1 LA: n = 54 (51,9%)	Multiple LA: n = 50 (48,1%)
LA Territory	II:51% III: 18,3% IV:2,9% V: 8,7%	
	Several territories: 19,2%	
Location of LA	Right: n = 61 (58,7%)	Left: n = 33 (31,7%)
	Bilateral: n = 10 (9,6%)	
LA Size	<3 cm: n = 36 (34,6%)	≥3 cm: 68(65,4%)
Abscess and/or fistula	n = 19 (18,3%)	
Duration of treatment	6 month: n = 80 (76,9%)	≥9 month: n = 24 (23,1%)
paradoxical upgrading reaction	n = 24 (23,1%)	
Resistance to antibacillaries	n = 12 (11,5%)	

n: number of patients, LA: Lymphadenopathy LNT: lymph node tuberculosis.

Patients were divided into 3 groups, group 1: patients with a single LA, group 2: patients with multiple LA, group 3: patients with abscess and/or external fistula, the results of treatment are presented in Table 2. Of the patients who had a single LA (n = 54), 26 had LA less than 3 cm, of which 11 patients did well on medical treatment alone, and 15 had residual LA after the end of treatment, for patients with LA greater than 3 cm (n = 28): 23 patients had LA after the end of treatment, and only 5 patients did well on medical treatment alone. For patients in group 2 (n = 50), 10 patients had LA less than 3 cm, of which 4 progressed well on medical treatment alone, while 6 kept residual LA, for the remaining 40 patients in this group (LA≥ 3 cm), 32 patients had kept LA after the end of treatment, while only 8 patients were cured on medical treatment alone. For the group with abscess and/or fistula, all patients received surgical treatment in addition to medical treatment (incision and drainage of the abscess, and excision of the fistula) except for one patient with external fistula, who received a fistula biopsy without excision in addition to antibacillary treatment, with good progression in 100% of cases. All the patients in the first two groups who kept lymphadenopathies after the end of the medical treatment have benefited from surgical treatment, either by an adenectomy in the case of a single lymphadenopathy, or lymph node dissection in the case of multiple lymphadenopathies.

**Table 2:** Treatment and evolution of our patients.

Characteristics of LA	LA Size		Treatment	Evolution	
				Cured	Not cured
Only one LA (n = 54)	<3 cm	n = 26 (48,2%)	Medical treatment	n:11(42,3%)	n:15(57,7%)
	≥3 cm	n = 28 (51,8%)	Medical treatment	n:5(17,9%)	n:23(82,1%)

Several LA (n = 50)	<3 cm	n = 10 (20%)	Medical treatment	n:04 (40%)	n:06 (60%)
	≥3 cm	n = 40 (80%)	Medical treatment	n:08 (20%)	n:32 (80%)

Abscess and/or Fistula (n = 19)	<3 cm	n = 9 (36%)	Drainage and/or Excision + Medical treatment	n:19 (100%)	n:0 (0%)
	≥3 cm	n = 16 (64%)			

n: number of patients, LA: Lymphadenopathy.

Among patients with documented resistance to antibacillary drugs (n = 12), only 1 patient responded to medical treatment alone, while 11 patients (91.7%) required surgery after failure of medical treatment. Of the patients who developed a paradoxical upgrading reaction after initiation of medical treatment (n = 24), 2 responded to medical treatment alone, and the remaining 22 patients required surgery after failure of medical treatment.

At the end of the study we performed a statistical analysis between patients who received medical treatment alone (n = 20; 19.2%), and patients who required surgery after failure of medical treatment (n = 84; 80.8%).

The causes of failure of medical treatment in LNT are illustrated in Table 3.

**Table 3:** Statistical analysis for the use of surgical treatment in lymph node tuberculosis.

Variables	Type of treatment		Univariate analysis			Multivariate analysis		
	*Medical*	*Medical and surgical*	OR	CI (95%)	P-value	OR	CI (95%)	P-value
LA size (≥3cm/<3 cm)	28 (29,9%)	76(73,1%)	3,022	1,233–7,410	0,014	6,813	1,795–25,860	0,005

LNT recurrence (n = 27)	3(11,1%)	24(88,1%)	3,846	1,057–13,994	0,043	8,606	1,762–42,040	0,008

Evolution period	-<2 month:		0,543	0,221–1,333	0,180	0,834	0,222–3,129	0,788
	12(11,55%)	22(21,2%)						
	-≥2 month							
	16(15,4%)	54(51,9%)						

Abscess/fistula (n = 19)	1(5,3%)	18(94,7%)	8,379	1,063–66,060	0,021	29,416	2,778–311,485	0,005

Resistance to treatment (n = 12)	1(8,3%)	11(91,7%)	4,569	0,562–37,154	0,174	16,648	1,281–216,337	0,032

paradoxical upgrading reaction (n = 24)	2(8,3%)	22(91,7%)	5,296	1,157–24,246	0,019	21,544	3362–138,069	0,001

Duration of medical treatment	−6 month:		0,314	0,086–1,152	0,113	0,376	0,070–2,032	0,256
	25(24%)	55(52,9%)						
	-≥9 month:							
	3(2,9%)	21(20,2%)						

OR: odds ratio CI (95%): 95% confidence interval n: number of patients.

In the univariate analysis, the size of LA≥3 cm (p = 0.014. OR = 3.022. IC = 1.233–7.410), recurrence of LNT (p = 0.043. OR = 3.846. IC = 1.057–13.994), presence of an abscess and/or fistula (p = 0.021. OR = 8379. CI = 1063–66,060), and the development of a paradoxical upgrading reaction (p = 0,019. OR = 5296. CI = 1157–24,246) were significantly correlated with the use of surgery. In the multivariate analysis, the size of LA≥3 cm (p = 0.005. OR = 6.813. IC = 1.795–25.860), recurrence of LNT (p = 0.008. OR = 8.606. IC = 1.762–42.040), presence of an abscess and/or fistula (p = 0.005. OR = 29.416. IC = 2.778–311.485), resistance to medical treatment (p = 0.032. OR = 5.296. IC = 1.157–24.246) were significantly correlated with the use of surgery. OR = 16,648 IC = 1281–216,337), and development of a paradoxical upgrading reaction (p = 0.001. OR = 21,544. IC = 3362–138,069) were significantly correlated with the use of surgery.

## 4 Discussion

In our study, out of a total of 104 patients, 84 patients (80.8%) required surgical treatment, and only 20 patients (19.2%) were reported cured under medical treatment alone, which is a high proportion compared to the study by Mohapatra et al., who operated on half of the patients (50.8%, 33) [10], and much higher than Patel who successfully treated 84% of patients in a prospective study of 130 cases with short-term chemotherapy for six months, But Patel has performed an adenectomy to establish the diagnosis, which is considered by us to be a surgical treatment [11].

Statistical analysis identified an association between LA size greater than or equal to 3 cm, tuberculosis recurrence, paradoxical upgrading reaction, antibacillary resistance, abscess and/or fistula, and the use of surgery in the treatment of cervical LNT.

Regarding LNT recurrence, 24 (23.1%) patients who had already been treated for LNT required surgery after the failure of medical treatment, which is similar to the study of Pallavi et al., who followed 91 patients with LNT on medical treatment alone, among these patients 12 (13%) recurred after the end of treatment, which motivated surgical treatment with good results [8]. While Omura et al., in a study of 38 LNT cases, found that 10 patients (26.3%) had already been treated for tuberculosis, all 10 patients responded well to medical treatment alone [12].

We found 12 patients with antibacillary resistance, 11(10.6%) patients benefited from lymph node dissection. Omura et al. [12], shares the same approach in their study by performing lymph node dissection in 3/38 patients with antibacillary resistance. Fontanilla et al. [13], in a review of the literature, recommend surgery whenever there is resistance to anti bacillaries.

The paradoxical upgrading reaction to antibacillary drugs is reported in 20–23% of HIV-negative patients [[14], [15], [16]], this reaction was found in 24 (23.1%) patients in our study, 22 (21.1%) patients were successfully operated on. This reaction is defined as an increase in the size of the LA, the appearance of new LA, a new fistula, in patients who received at least 10 days of treatment [15]. According to an english study, it occurs at a median of 46 days after the beginning of treatment [15]. There are two explanations for this reaction: a delayed immune reaction, or hypersensitivity to the antigens released by lysed mycobacteria, some authors report a beneficial effect of corticosteroids [17,18], but retrospective studies have shown that corticosteroids did not prevent the paradoxical upgrading reaction in patients who had already started treatment [19], and had no effect on its duration [14,15], in our study we did not use corticosteroids in any of our patients. The paradoxical upgrading reaction requires prolonged antibiotic therapy with unsatisfactory results. Several authors recommend surgery in this case to avoid these poor results [12,15].

For LA greater than or equal to 3 cm, Kanjanopas et al., found a 100% cure rate after complete surgical excision of LA≥3 cm before receiving medical treatment [20]. Another study conducted by Sui et al., reported a 100% cure rate after excision of all LA≥3 cm [21]. Other authors report the usefulness of surgery, as it allows rapid tissue diagnosis [[22], [23], [24]], and increases the cure rate with an excellent aesthetic result and a low complication rate [25].

Among the limitations of the present study were the lack of systematic use for all patients of the gold standard for the diagnosis of tuberculosis, which is culture, which allows bacteriological confirmation, as well as the study of resistance to antibacillary drugs, other limitations of this study were the absence of the immune status of the patients, and the use of lymph node biopsy, which is the most invasive approach for the diagnosis of LNT.

## 5 Conclusion

Antibacillary chemotherapy is the main treatment for lymph node tuberculosis. Based on our experience, we recommend excision of adenopathies ≥3 cm in diameter, abscesses, and fistulas. Lymph node dissection is also indicated in case of recurrence, resistance to antibacillary drugs, and paradoxical upgrading reaction. Early surgical intervention in these patients reduces complications such as the spread of the disease to other organs, reduces morbidity and improves the quality of life of patients.

Application of these outcomes in other works is required to evaluate it, and to propose modifications to these recommendations.

## Funding

Funding sources: This research did not receive any grant or funding from governmental or private sectors.

## Author contribution

Lekhbal Adil: Corresponding author, writing the paper.

Chakir kaoutar: writing the paper.

Halily sara: writing the paper.

Reda Abada: correction of the paper.

Sami Rouadi: study concept.

Mohamed Roubal: study concept.

Mohamed Mahtar: correction of the paper.

## Registration of research studies

Researchregistry5424

## Guarantor

Lekhbal Adil.

## Provenance and peer review

Not commissioned, externally peer reviewed.

## Declaration of competing interest

All authors have no conflict of interest or financial support with this article.
