# Supplementary material for: Treatment of cervical lymph node tuberculosis: When surgery should be performed? A retrospective cohort study
Source: Ann Med Surg (Lond). 2020 May 18;55:159–63. doi: 10.1016/j.amsu.2020.05.006 (PMC7256428; doi:10.1016/j.amsu.2020.05.006)
Supplement: Multimedia component 1 [file mmc1.docx]

| The STROCSS 2019 Guideline | | |
| --- | --- | --- |
| Item no. | **Item description** | **Page** |
| TITLE | | |
| 1 | **Title**: Treatment of cervical lymph node tuberculosis: when surgery should be performed? a retrospective cohort study | 1 |
| ABSTRACT | | |
| 2a | **Introduction**: lymph node tuberculosis is the most common form of extra pulmonary tuberculosis. Although diagnosis is usually difficult, therapeutic management remains a challenge and a subject of national and international debate. | 1 |
| 2b | **Methods**: a retrospective cohort study  the medical records of patients with cervical lymph node tuberculosis who were treated at 20 August Hospital, Casablanca, Morocco, between May 2017 and November 2018 were reviewed. The results of the treatment were analyzed. | 1 |
| 2c | **Results**: Out of a total of 104 patients, the mean age was 24 years, the sex ratio was 0.51 (women: 66.3%), twenty (19.2%) patients received medical treatment alone, and 84 (80.8%) patients required medical and surgical treatment. Surgery was required when the size of the lymphadenopathies was greater than or equal to 3cm (p=0.005), when the patient presented with an abscess and/or fistula(p=0.005), when the patient presented with resistance to antibacillary drugs(p=0.032), or developed a paradoxical upgrading reactions (p=0.001), or when the patient had a recurrence of lymph node tuberculosis(p=0.008) on multivariate analysis. | 1 |
| 2d | **Conclusion**: antibiotic therapy remains the main treatment for all patients with lymph node tuberculosis, but the results of our work show the value of surgery in some indications. | 1 |
| INTRODUCTION | | |
| 3 | Tuberculosis (TB) is a major public health problem in the international community, with the World Health Organization (WHO) estimated at 10.4 million cases in 2016 [1]. Extra pulmonary tuberculosis (EPTB) does not exceed 15% of all TB patients reported for many years [1, 2]. However, there are some differences between WHO regions.  In Morocco, WHO estimates for the year 2016 that approximately 36,000 people have been affected by tuberculosis, with an estimated incidence of 103 cases per 100,000 persons [1]. Between 1980 and 2016, the proportion of reported EPTB cases increased from 23% to 46%, while the proportion of pulmonary TB cases decreased from 63% to 54% [3]. lymph nodes tuberculosis (LNT) is the most common form of EPTB, accounting for 37% of all reported EPTB cases in Morocco in 2016 [3].  Currently, LNT is treated with multidrug antibacillary chemotherapy [4]. However, in the 1970s, surgery was often necessary in the treatment of LNT , as the chemotherapy used, based on streptomycin, isoniazid, and paraaminosalicylate (PAS) for 3 years, did not give satisfactory results [5]. For this reason, ENT surgeons preferred surgery without medical treatment until the early 1990s [6], after which the WHO recommended treatment with rifampicin and isoniazid for 6 months and pyrazinamide for 2 months (WHO, 1997), this treatment was adjusted over the years by the WHO, and the number of publications regarding surgery for LNT has decreased considerably.  The Infectious Diseases Society of America (IDSA) recommends surgery for LNT only in unusual circumstances, but these are not explicit [7]. Although surgical excision combined with antibiotic therapy produces favorable results. We are not aware of any controlled studies that have compared the results of medical and surgical treatment with medical treatment alone [8].  In our context we find ourselves more and more in situations where patients retain some lymphadenopathies, or develop others despite well-conducted medical treatment.  The main goal of our stady was to identify causes of the failure of medical treatment in LNT, and to propose indications for the use of surgery in cervical LNT in Morocco, and the secondary goal was to identify the epidemiological and clinical characteristics of our patients. | 3 |
| METHODS | | |
| 4a | **Registration and ethics**:  researchregistry5424 |  |
| 4b | **Ethical Approval**: Not required |  |
| 4c | **Protocol:**  Patients with cervical LNT confirmed on lymph node biopsy were followed  study has compared patients who were reported to be cured under medical treatment alone after a lymph node biopsy, and patients who required surgery after failure of medical treatment | 4 |
| 4d | **Patient Involvement in Research**  we have reviewed the patients in a retrospective view, comparing those who have successfully managed under medical treatment alone and those who needed surgery. | 4 |
| 5a | **Study Design:**  Cohort study  Design: retrospective, single centred) | 4 |
| 5b | **Setting**: the following areas are described comprehensively  Geographical location: Morocco  Nature of institution:public  Dates: period of 18 months (May 2017 to November 2018). | 4 |
| 5c | **Cohort Groups**:  Number of groups: 2 groups  Division: who were reported to be cured under medical treatment alone after a lymph node biopsy, and patients who required surgery after failure of medical treatment. | 4 |
| 5d | **Subgroup Analysis**:  Patients were divided into 3 groups according to Lymphadenopathy (LA) characteristics (number of LA, as well as their size, presence of an abscess and/or fistula). Regarding the size of the LA, a diameter of 3 cm is used as a reference to differentiate between patients. | 4 |
| 6a | **Participants**:  Patients with cervical LNT confirmed on lymph node biopsy, by the presence of granulomatous inflammation with caseous necrosis, and/or Xpert MTB/RIF positivity with study of rifampicin resistance, and/or positive culture with study of antibacillary resistance, were included in the study. Patients received antibacillary multidrug therapy based on WHO recommendations in the treatment of LNT. While patients undergoing first-line surgical treatment outside of abscess and fistula, and those with poor adherence to medical treatment were excluded from the study.  In this study, " cure " was defined as the complete disappearance of LA, abscesses, and fistulas after at least 6 months after the end of treatment | 4 |
| 6b | **Recruitment**:  After failure of medical treatment  18 onth | 4 |
| 6c | **Sample Size**:  p<0.05 was retained as the significant threshold with a 95% confidence interval (CI)  The statistical analysis was performed with SPSS 23 software (SPSS Inc., Chicago, IL, USA), the statistical tests used were Chi-2 (or Fisher test when the theoretical counts were less than or equal to 5%) for the analysis of nominal variables, the analysis of ordinal variables, and quantitative variables was performed by Spearman correlations | 4 |
| INTERVENTION AND CONSIDERATIONS | | |
| 7a | **Pre-intervention Considerations:**  Patients with a confirmed diagnosis of tuberculosis of the lymph nodes have been put on antibacillary treatment according to WHO recommendations. | 4 |
| 7b | **Intervention**:  After medical treatment failed, the patients were treated surgically. In case of single adenopathy, an adenectomy was performed, in case of multiple adenopathies a modified lymph node dissection was performed, and in case of abscesses or fistula, an incision with drainage of the abscess or excision of the fistula was performed. | 4 |
| 7c | **Intra-Intervention Considerations**: in case of a single adenopathy an adectomy under local anaesthesia has been performed, and in case of multiple adenopathies a modified lymph node dissection under general anaesthesia has been performed | 4 |
| 7d | **Operator Details:**  No Training needed | 4 |
| 7e | **Quality Control**:  to reduce variation only one surgical technique is performed by all surgeons | 4 |
| 7f | **Post-Intervention Considerations**:  In this study, " cure " was defined as the complete disappearance of LA, abscesses, and fistulas after at least 6 months after the end of treatment | 4 |
| 8 | **Outcomes**:  the primary results concern the epidemiology of our patients and the clinical characteristics, after division of the patients into subgroups an analysis was made to reveal the causes of the failure of the medical treatment. | 4 |
| 9 | **Statistics**:  The statistical analysis was performed with SPSS 23 software (SPSS Inc., Chicago, IL, USA), the statistical tests used were Chi-2 (or Fisher test when the theoretical counts were less than or equal to 5%) for the analysis of nominal variables, the analysis of ordinal variables, and quantitative variables was performed by Spearman correlations, A p<0.05 was retained as the significant threshold with a 95% confidence interval (CI). A multivariate analysis by logistic regression was then conducted, taking as explained variabIe: "the use of surgical treatment after failure of medical treatment" and as explanatory variables ceIIes whose degree of significance was less than p<0.20 in univariate anaIysis, from this initial muItivariate model, the variabIes whose adjusted degree of significance remained less than p<0.05 were kept in the final model.  Sub-group analysis: Patients were divided into 3 groups according to Lymphadenopathy (LA) characteristics (number of LA, as well as their size, presence of an abscess and/or fistula). Regarding the size of the LA, a diameter of 3 cm is used as a reference to differentiate between patients. | 4-5 |
| RESULTS | | |
| 10a | **Participants**: During the study period, 104 cases of LNT were included in the study, the sex ratio was 0.51( women:66%), the mean age was 24 years, the age range between 20 and 40 years accounted for 51% of the cases, 27 patients had a recurrence of LNT, while 7 patients had multifocal tuberculosis, the duration of symptom progression was less than 2 months in 32.7% of the cases, and more than 2 months in 67.3% of the cases. Clinically, a single LA was found in 51.9% of cases, and multiple LA in 48.1% of cases, an abscess and/or fistula in 19 patients, the size of the LA was less than 3 cm in 34.6% of cases, and greater than or equal to 3 cm in 65.4% of cases, Resistance to antibacillaries was found in 12 (11.5%) patients, the duration of medical treatment was 6 months in 76.9% of cases, and greater than or equal to 9 months in 23.1% of cases, during medical treatment, 24 (23.1%) patients developed a paradoxical upgrading reaction to antibacillaries | 5 |
| 10b | **Participant Comparison**:  Table comparing demographics included | 12-13 |
| 10c | **Intervention**: Patients were divided into 3 groups, group 1: patients with a single LA, group 2: patients with multiple LA, group 3: patients with abscess and/or external fistula, the results of treatment are presented in | 5 |
| 11a | **Outcomes**: Of the patients who had a single LA (n=54), 26 had LA less than 3 cm, of which 11 patients did well on medical treatment alone, and 15 had residual LA after the end of treatment, for patients with LA greater than 3 cm (n=28): 23 patients had LA after the end of treatment, and only 5 patients did well on medical treatment alone. For patients in group 2 (n=50), 10 patients had LA less than 3 cm, of which 4 progressed well on medical treatment alone, while 6 kept residual LA, for the remaining 40 patients in this group (LA≥ 3 cm), 32 patients had kept LA after the end of treatment, while only 8 patients were cured on medical treatment alone. For the group with abscess and/or fistula, all patients received surgical treatment in addition to medical treatment (incision and drainage of the abscess, and excision of the fistula) except for one patient with external fistula, who received a fistula biopsy without excision in addition to antibacillary treatment, with good progression in 100% of cases. All the patients in the first two groups who kept lymphadenopathies after the end of the medical treatment have benefited from surgical treatment, either by an adenectomy in the case of a single lymphadenopathy, or lymph node dissection in the case of multiple lymphadenopathies. | 5-6 |
| 11b | **Tolerance**: In our study we successfully monitored 104 patients, for those lost to follow-up, we noted 26 patients. | 4 |
| 11c | **Complications**: We didn't find any complications after the surgery | 5 |
| 12 | **Key Results**: In the univariate analysis, the size of LA≥3cm (p=0.014. OR=3.022. IC=1.233-7.410), recurrence of LNT (p=0.043. OR=3.846. IC=1.057-13.994), presence of an abscess and/or fistula (p=0.021. OR=8,379. CI=1,063-66,060), and the development of a paradoxical upgrading reaction (p=0,019. OR=5,296. CI=1,157-24,246) were significantly correlated with the use of surgery. In the multivariate analysis, the size of LA≥3cm (p=0.005. OR=6.813. IC=1.795-25.860), recurrence of LNT (p=0.008. OR=8.606. IC=1.762-42.040), presence of an abscess and/or fistula (p=0.005. OR=29.416. IC=2.778-311.485), resistance to medical treatment (p=0.032. OR=5.296. IC=1.157-24.246) were significantly correlated with the use of surgery. OR=16,648 IC=1,281-216,337), and development of a paradoxical upgrading reaction (p=0.001. OR=21,544. IC=3,362-138,069) were significantly correlated with the use of surgery. | 6 |
| DISCUSSION | | |
| 13 | **Discussion**:  According to the results of our work we recommend to operate patients with lymph node tuberculosis in case of: adenopathies ≥3 cm in diameter, abscesses, and fistulas, recurrence, resistance to antibacillary drugs, and paradoxical upgrading reaction.  The majority of studies reviewed in the literature share the same conduct except that we have not found any studie that treat the subject in its integrality. on the other hand, the percentage of patients who required surgery in our study is higher than in other studies | 6-7-8 |
| 14 | **Strengths and Limitations:**  Strengths of the study: We are not aware of any controlled studies that have compared the results of medical and surgical treatment with medical treatment alone.  Limitations: Among the limitations of the present study were the lack of systematic use for all patients of the gold standard for the diagnosis of tuberculosis, which is culture, which allows bacteriological confirmation, as well as the study of resistance to antibacillary drugs, other limitations of this study were the absence of the immune status of the patients, and the use of lymph node biopsy, which is the most invasive approach for the diagnosis of LNT. | 8 |
| 15 | **Implications and Relevance:**  we recommend excision of adenopathies ≥3 cm in diameter, abscesses, and fistulas. Lymph node dissection is also indicated in case of recurrence, resistance to antibacillary drugs, and paradoxical upgrading reaction.  prospective work is needed in this direction to evaluate these results. | 6-7-8 |
| CONCLUSION | | |
| 16 | Antibacillary chemotherapy is the main treatment for lymph node tuberculosis. Based on our experience, we recommend excision of adenopathies ≥3 cm in diameter, abscesses, and fistulas. Lymph node dissection is also indicated in case of recurrence, resistance to antibacillary drugs, and paradoxical upgrading reaction. Early surgical intervention in these patients reduces complications such as the spread of the disease to other organs, reduces morbidity and improves the quality of life of patients.  Application of these outcomes in other works is required to evaluate it, and to propose modifications to these recommendations. | 8 |
| DECLARATIONS | | |
| 17a | **Conflicts of interest**  All authors have no conﬂict of interest or ﬁnancial support with this article. | 9 |
| 17b | **Funding**  Funding sources this research did not receive any grant or funding from governmental or private sectors. | 9 |
